# Sex Differences in Attitudes Toward Casual Sex: Using STI Contraction Likelihoods to Assess Evolved Mating Strategies

**DOI:** 10.3389/fpsyg.2021.706149

**Published:** 2021-09-03

**Authors:** R. Nathan Pipitone, Lesley Cruz, Helen N. Morales, Daniela Aladro, Serena R. Savitsky, Maria Koroleva, Francesca Valdez, Erin Campbell, Sam Miranda

**Affiliations:** ^1^Department of Psychology, Florida Gulf Coast University, Fort Myers, FL, United States; ^2^Department of Psychology, University of Stirling, Stirling, United Kingdom

**Keywords:** casual sex, evolved mating strategies, sexually transmitted infections, sex differences, sociosexuality, risk-taking

## Abstract

Previous work shows that males are more likely to pursue casual sex if given the opportunity, compared to females, on average. One component of this strategy is risk-taking, and males have been shown to take more risks than females in a variety of contexts. Here, we investigate the extent to which sex differences exist considering casual sexual encounters involving sexually transmitted infections (STIs) using a hypothetical sexual scenario which attempts to circumvent several factors that may contribute to a female’s hesitancy to engage in casual sex encounters. Two hundred and forty-six college students rated their willingness to engage in a satisfying casual sexual encounter with someone judged to be personable as a function of sex, varying STI contraction likelihoods, several STI types, and two levels of hypothetical partner attractiveness. We also assess how individual levels of sociosexuality (as measured by the SOI-R) impact findings. Our findings show that males report higher likelihoods of sexual engagement compared to females in general. This trend continued for lower likelihoods of STI contraction in all four STI types (Cold, Chlamydia, Herpes, HIV), with larger effects shown in the high attractiveness partner condition. For higher STI contraction likelihoods and more severe STI types, along with lower partner attractiveness levels, sex differences shrank. Factoring in participant SOI-R scores attenuated the effects somewhat, although it failed to alter findings substantially with predicted sex differences continuing to exist. These results offer further insight into evolved sex differences in human mating systems and provide an additional framework to test sexual risk-taking among males and females.

## Introduction

Over the last 50 years, scientists interested in the evolutionary underpinnings of sex differences in mating strategies have utilized Triver’s (1972) theory of parental investment, which states that the sex that invests more in offspring (physiologically and/or behaviorally) will be more selective when it comes to choosing a mate, with the sex investing less competing for access to mates. In 1979, Donald Symons further explicated theoretical reasoning for males being more likely to pursue sexual variety under an evolutionary framework. Among humans, although both males and females pursue long- and short-term mating scenarios under different ecological conditions (Gangestad and Simpson, 2000; Buss, 2006; Buss and Schmitt, 2019), divergent investment strategies between males and females exist. Several lines of evidence show that males are on average more likely to prefer sexual variety and pursue more opportunistic mating strategies, compared to females (Clark and Hatfield, 1989; Clark, 1990; Kenrick et al., 1990; Simpson and Gangestad, 1992; Buss and Schmitt, 1993; Schmitt, 2003; Guéguen, 2011). Even when intentionally pursuing casual sex encounters, females show different emotional reactions to those events compared to males, such as having less permissive attitudes and are more likely to experience worry-vulnerability (Townsend and Wasserman, 2011; Townsend et al., 2015) and lower well-being (Mafra et al., 2021). While some work has questioned the above findings (e.g., Conley et al., 2011), careful examination of the issues and considering actual mating decisions reveal these differences to be largely robust (Schmitt et al., 2012). Frederick et al. (2019) recently conducted a large-scale study and concluded that sex differences in short-term mating encounters do exist, even when considering partner mediating factors such as social status, athleticism, and resources. Along with male’s preference for sexual variety, mate quality characteristics such as personality attributes (Buss and Schmitt, 1993; Surbey and Conohan, 2000) are judged to be not as important for males in short-term mating encounters, compared to females, although males continue to consider attractiveness in these decisions (Surbey and Conohan, 2000; Todd et al., 2007; Schützwohl et al., 2009).

One component of pursuing short-term mating scenarios and sexual variety is risk-taking. While it is the case that both sexes incur more risk under short-term mating scenarios (Sylwester and Pawłowski, 2011) and certain environmental factors (i.e., alcohol consumption) can increase risk-taking in both sexes (George et al., 2008), the evidence to date shows that younger males take on a more risky life history strategy (Daly and Wilson, 1988; Kruger, 2008; Wang et al., 2009) with males willing to incur more risk than females in a variety of contexts (e.g., Byrnes et al., 1999; Courtenay, 2000; Waldron et al., 2005; George et al., 2008; Pawłowski et al., 2008; Johnson et al., 2017). Males will increase their risk-taking when in the presence of an attractive female (Ronay and von Hippel, 2010) or when presented with attractive female face stimuli (Baker and Maner, 2008). What is more, risk-taking males are found to be attractive to females under short-term mating scenarios (Li and Kenrick, 2006; Vincke, 2016).

Focusing on risky behaviors among college students, Rolison and Scherman (2003) show that males report more frequent risky behaviors overall, with fewer perceived risks and more benefits from displaying such behaviors. A largely unexplored area among this population is the risk of contracting a sexually transmitted infection (STI). Earlier work shows that the sexual behavioral patterns of males puts them at a higher-risk for contracting HIV and other STIs, with STI infection risk being double for males than females (Courtenay, 2000). Although previous work shows that females are more biologically susceptible to contracting STIs in cases where transmission is from male to female (Panchanadeswaran et al., 2006), recent cases in the US show males are five times more likely to contract HIV (U.S. Department of Health and Human Services, 2021). In 2018, it was reported that half of the 20 million STI cases in the US were contracted among youth aged 15–24 (Shannon and Klausner, 2018) with similar rates of susceptibility overall among both sexes in 2021 (although this varied by infection type) (Kreisel et al., 2021). Therefore, STIs pose a threat to the health of college students, and within the context of the present study, the higher likelihood of engaging in a sexual encounter with someone that has an STI would be a direct measurement of their risk-taking.

### Present Study

The goal of the current study is to assess the extent to which sex differences in hypothetical short-term mating scenarios exist when considering a form of risk-taking: STI contraction likelihood, and STI type. We also explore how levels of attractiveness (hypothetical partner) impact these findings.

In an attempt to limit sex-biased responses to short-term mating scenarios, our methodology provides important contextual cues to minimize potential confounds that may lead to sex differences in pursuing short-term mating, for example, concerns over pregnancy, sexual gratification, and lack of social context/assessment (Conley et al., 2011; Pedersen et al., 2011; Galperin et al., 2013; Kennair et al., 2016) although some work (Clark, 1990) has previously shown that lack of personal safety does not play a role. Specifically, male and female college students will be provided with hypothetical scenarios and rate their willingness to engage in casual sex with someone whom they have met at a party. The hypothetical scenario involves having a great conversation with the person they have met, and this person has been judged to be personable and interested in them socially. The hypothetical scenario will involve a satisfying sexual experience (with no possibility of pregnancy). Likelihoods of STI contraction and STI types will vary across the different conditions. Considering the 0% of STI contraction, we predict to find smaller differences in sexual engagement likelihoods between males and females, because of the scenario details previously mentioned. However, as STI contraction likelihoods and STI severity increase, we predict that sex differences in rater’s responses will manifest to reflect male’s more opportunistic/risky behavioral repertoires. However, as contraction likelihoods increase to higher thresholds (e.g., 50 and 100%), we predict that males and females will again resort to more similar rates of sexual engagement likelihoods, particularly for the more severe STI types. We will also explore how attractiveness levels of the hypothetical partner play a role in participants’ responses.

In addition, we will explore how levels of sociosexuality impact findings. The SOI (Simpson and Gangestad, 1991) and the revised SOI-R (Penke and Asendorpf, 2008) can be used as a metric for one’s behavior, attitude, and desire related to uncommitted sexual experiences, with Likert scale responses to items such as “Sex without love is OK,” and “How often do you have fantasies about having sex with someone you are not in a committed romantic relationship with?” Males have shown to be less restricted across a variety of cultures (Schmitt, 2005; Lippa, 2009) and have higher SOI-R values compared to females (e.g., Penke and Asendorpf, 2008; Kennair et al., 2016; Nascimento et al., 2018). Some work has shown that by controlling for SOI-R, sex differences considering short-term mating scenarios are mitigated (Hallam et al., 2018). While this may be the case, other work (Kennair et al., 2016) questions whether this tactic is justified, since controlling for levels of sociosexuality, which inherently taps into the constructs of “maleness” and “femaleness,” will shrink any sex difference in mating behavior one is trying to investigate. In the present study, we investigate the extent to which SOI-R levels impact attitudes toward risky casual sex encounters among males and females.

## Materials and Methods

### Participants

Two hundred and forty-six individuals participated in the study. All the participants were undergraduate students from a Southeastern university in the United States. They were recruited from the institution’s General Psychology research pool (Sona-Systems^®^)^1^ for class credit or from other classrooms for extra credit. The study was approved by the institution’s IRB (protocol #2019–20). The sample consisted of 111 males (45%), and 135 females (55%). Seventy two percent were Freshmen, 21% were Sophomores, 5% were Juniors and 2% were Seniors. Participant self-identified race categories were as follows: One hundred and fifty-seven (64%) were White (Caucasian), 38 (15%) were Hispanic/Latino, 17 (7%) were Black (e.g., African/Caribbean American), 5 (2%) were Asian/Pacific Islander, 28 (11%) indicated being biracial, and 1 person (less than 1%) indicated being Other. Two hundred and five (84%) participants identified as being heterosexual, 24 (10%) identified as bisexual, 13 (5%) identified as homosexual, and 3 (1%) as other, with 1 participant not providing a response. Three individuals identified as transgender (female-to-male). The inclusion or exclusion of their responses did not impact findings, hence were included in all analyses as males. Responses from homosexual individuals and those indicating other orientations (e.g., bisexual) did not significantly impact findings, hence were included in all analyses. See Table 1 for descriptive statistics on the additional demographic variables collected in the study. Several of these variables were found to be significantly different between males and females. None of these variables impacted the overall findings when entered into the model as covariates.

**TABLE 1:** Descriptive statistics of demographic information collected from participants in the study.

Variable	Males (*N* = 111)	Females (*N* = 135)
Age*	19.17 (2.55)	18.57 (1.17)
Relationship status*		
Single	70%	56%
In a relationship	30%	44%
Number of sexual partners	4.81 (4.96)	3.97 (5.14)
SOI-R composite score**	24.63 (7.84)	20.22 (6.78)
Previous sex education course	74%	76%
Religious affiliation	53%	53%
Currently sexually active	68%	74%
Previous STI	1%	5%

STI = sexually transmitted infection. Variables with averages have standard deviations given in parentheses. Percentages indicate the number of participants responding yes to the question. Significant differences between males and females are indicated as follows: *p < 0.05. **p < 0.001.

### Procedure

#### Participants

Participants were brought into a research lab by themselves and sat in front of a computer to complete the study. The online survey consisted of several sections. The first section included demographic questions such as age, sex, sexual orientation, religiosity, current relationship status, history of any sexually transmitted infections, and sex education history (see Table 1 for descriptive results).

#### Sociosexuality Section

In the second section, participants filled out the revised Sociosexual Orientation Inventory (SOI-R) adapted from Penke and Asendorpf (2008). The SOI-R captures sociosexuality or sociosexual orientation, as well as individual differences in the likelihood to engage in an uncommitted, casual sexual relationship. Scores were aggregated from the 9-item measure. Internal consistency was good for responses overall (Cronbach’s alpha = 0.86). See Table 1 for descriptive results.

#### STI Section

The third section included a brief description of the different STI types used in the study (Common Cold, Herpes, Chlamydia, and HIV). This included infection symptomatology, treatment options, and outcome if left untreated. See Supplementary Datasheet 1 for descriptions of each. This was done to ensure participants had some basic knowledge about the different types of STIs and the severity of each. We also had participants rank each STI from least to most severe, see results section for details.

#### Hypothetical Sexual Scenario

In the fourth section, participants were asked to consider the following scenario and to answer truthfully:

*“Imagine you are single and someone approaches you and you start talking with them and you end up hitting it off immediately and have a great conversation. This person says they are very interested in you and would like to pursue a brief satisfying sexual encounter (sexual intercourse). In each scenario, consider yourself having unprotected sex with no chance of pregnancy.”*

The dependent variable measured in the study was Sexual Engagement Likelihood (0–100 sliding scale). Participants were asked to indicate how likely they would engage in a sexual encounter based on several different situations, or variables used in the study. One variable was STI Contraction Likelihood (0, 5, 25, 50, and 100%). A second variable was STI Type (Common Cold, Herpes, Chlamydia, and HIV). A third variable was hypothetical partner Attractiveness Level (7 or 10 on a scale of 1–10). Below are two examples of questions utilizing the different variable conditions:

1. *“This person is a 7 on a scale of 1–10 (10 being very attractive). You have a 5% chance of contracting a common cold from this person. Use the slider to indicate how likely you would be to engage in this sexual encounter with this person?”*
2. *“This person is a 10 on a scale of 1–10 (10 being very attractive). You have a 50% chance of contracting HIV from this person. Use the slider to indicate how likely you would be to engage in this sexual encounter with this person?”*

## Results

### STI Severity Analyses

Participants ranked the STIs used in the study on levels of severity. The Common Cold was ranked as least severe by 92% of participants. Ninety percent of participants ranked HIV as the most severe. Chlamydia was ranked second least severe by 47% of participants (43% ranked Herpes as second least severe) and 53% of participants ranked Herpes as second most severe (41% ranked Chlamydia as second most severe). Males and females had similar rankings of STI severity (all *X*^2^ values < 6.5, *p’s* > 0.09. See Supplementary Table 4 for details.

G^∗^Power 3.1.9.7 was used estimate power for the most stringent test conducted (the mixed-model ANOVAs with the variable Sex as a between-subjects factor and the removal of 20 participants because of their 0% sexual engagement likelihood across all conditions). Using a medium effect size estimation and alpha of.05, degrees of freedom of (12,213), the calculated power was 99%.

### Main Analyses

Statistical tests were calculated using the General Linear Model (GLM) procedure in SPSS^®^ version 26 (IBM, United States). The repeated measures GLM procedure provides profile analysis results which uses a multivariate approach to analyze repeated measures data when sphericity is an issue (Tabachnick et al., 2007, p. 330). Hence, we interpreted the multivariate *F*-tests (Pillai’s Trace) throughout. However, interpreting the univariate results using the adjusted statistics when sphericity was present (e.g., Greenhouse-Geisser correction) did not change any significance outcome in the study. A 2 (Sex) × 2 (Attractiveness Level) × 4 (STI Type) × 5 (STI Contraction Likelihood) mixed-model ANOVA was conducted as the overall interaction test to determine whether sex differences exist in how participants rated their Likelihood of Sexual Engagement across different STI Contraction Likelihoods, different STI Types, and Attractiveness Levels of the hypothetical partner. All variables were within-subjects except the variable, Sex. The four-way interaction was significant [*F*(12, 233) = 2.26, *p* = 0.01, η^2^ = 0.11]. Lower STI Contraction Likelihoods, less severe STI Types, and higher Attractiveness Levels in the hypothetical partner led to larger sex differences in Sexual Engagement Likelihood. As STI Contraction Likelihoods and STI Type severity increased, and Attractiveness Level decreased, sex differences shrank. See Table 2 for all main effects and interaction results.

**TABLE 2:** All mixed-model ANOVA main effects and 2-way, 3-way, and 4-way interactions of Sex, STI Contraction Likelihood, STI Type, and Attractiveness Level on the dependent variable, Sexual Engagement Likelihood. Bolded results represent targeted analyses, with the interactions of Sex * STI Contraction Likelihood and Sex * STI Contraction Likelihood * STI Type displayed in Figures 1, 2, respectively.

Independent Variables	*df_1_, df_2_*	*f*	*p*	η*^2^*
**Sex**	**1, 244**	**51.75**	**<0.001**	**0.18**
STI contraction likelihood	4, 241	213.76	<0.001	0.78
STI type	3, 242	142.35	<0.001	0.64
Attractiveness level	1, 244	192.61	<0.001	0.44
Sex * STI contraction likelihood	**4, 241**	**12.34**	**<0.001**	**0.17**
Sex * STI type	3, 242	15.33	<0.001	0.16
Sex * attractiveness level	1, 244	11.41	0.001	0.05
STI Type * attractiveness level	4, 241	46.92	<0.001	0.37
STI contraction likelihood * STI Type	12, 233	30.79	<0.001	0.61
STI contraction likelihood * attractiveness level	4, 241	17.71	<0.001	0.23
Sex * STI contraction likelihood * STI type	**12, 233**	**5.12**	**< 0.001**	**0.21**
Sex * STI Type * attractiveness level	3, 242	5.29	0.002	0.06
Sex * STI contraction likelihood * attractiveness level	4, 241	2.78	0.028	0.04
STI contraction likelihood * STI type * attractiveness level	12, 233	5.68	<0.001	0.23
Sex * STI contraction likelihood * STI type * attractiveness level	**12, 233**	**2.26**	**0.01**	**0.11**

**STI, Sexually Transmitted Infection; df_1_, numerator degrees of freedom; df_2_, denominator degrees of freedom; f, multivariate Pillai’s trace value; p, significance value (p < 0.05 considered significant); η^2^, partial eta-squared. The bolded values represent our target analyses performed in the study.**

**FIGURE 1:**
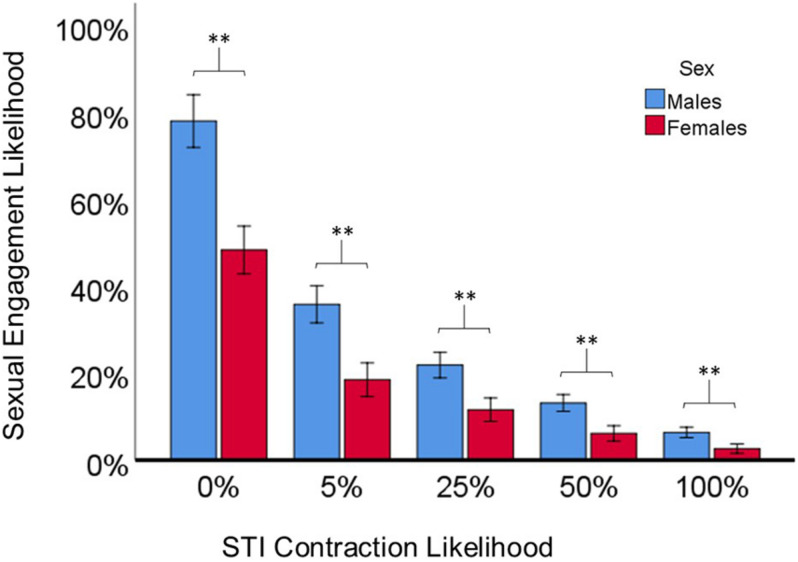
The significant two-way interaction of Sex and STI Contraction Likelihood on Sexual Engagement Likelihoods. Planned comparison *t-*tests show significant differences between males and females for all Contraction Likelihood conditions. Error bars represent 95% confidence intervals. ^∗∗^ indicates *p* < 0.001.

Figure 1 shows the two-way interaction Sex and STI Contraction Likelihood on Sexual Engagement Likelihoods, collapsed across different STI Types and Attractiveness Levels, [*F*(4, 241) = 12.34, *p* < 0.001, η^2^ = 0.17]. Independent sample *t*-tests were used to analyze the *a priori* planned comparisons of whether males and females differed in their Sexual Engagement Likelihood across the five different STI Contraction Likelihood scenarios. Males reported significantly higher Sexual Engagement Likelihoods compared to females across the five STI Contraction Likelihood scenarios (see Figure 1).

**FIGURE 2:**
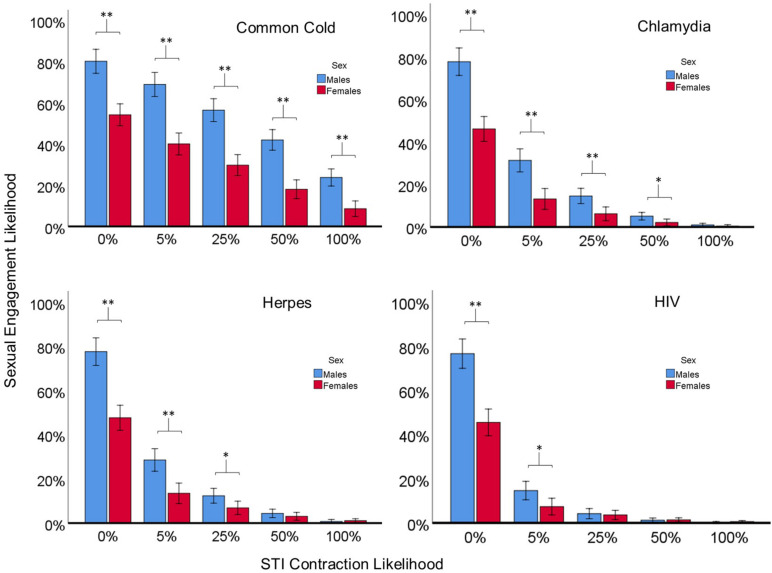
The significant three-way interaction of Sex, STI Contraction Likelihood, and STI Type on Sexual Engagement Likelihoods. Planned comparison *t-*tests conducted for each STI Type revealed significant differences in Sexual Engagement Likelihoods between males and females. Lower STI Contraction Likelihoods and less severe STI Types led to larger sex differences in Sexual Engagement Likelihood, and as STI Contraction Likelihoods and STI Type severity increased, sex differences shrank. Error bars represent 95% confidence intervals. ^∗∗^ indicates *p* < 0.001, ^∗^ indicates *p* < 0.05.

Figure 2 shows the three-way interaction of Sex, STI Contraction likelihood, and STI Type on Sexual Engagement Likelihoods, collapsed across Attractiveness Level [*F*(12, 233) = 5.12, *p* < 0.001, η^2^ = 0.21]. Lower STI Contraction Likelihoods and less severe STI Types led to larger sex differences in Sexual Engagement Likelihood, and as STI Contraction Likelihoods and STI Type severity increased, sex differences shrank. Independent sample *t-*tests were also used to analyze the planned comparisons of Sex across the five different STI Contraction Likelihood scenarios for each of the four different STI Types and can be seen in Figure 2. Descriptive and inferential statistics for all planned comparisons for all conditions can be found in Supplementary Table 1.

### SOI-R Analysis

To investigate whether participant’s level of sociosexuality impacted the above findings, participant’s composite SOI-R scores were entered into the GLM mixed-model ANOVA as a covariate. With SOI-R scores entered, several main effects and interactions that were not central to our main investigation had non-significant results. See Supplementary Table 2 for all ANCOVA results. SOI-R scores negated the effect that Attractiveness Level had on Sexual Engagement Likelihoods, therefore most models considering Attractiveness Level as a variable became non-significant. The 4-way interaction of Sex, STI Contraction Likelihood, STI Type, and Attractiveness Level on Sexual Engagement Likelihood was also no longer significant [*F*(12, 232) = 1.58, *p* = 0.097, η^2^ = 0.08], however, the change in effect size (η^2^) from the original 4-way interaction to the model with SOI-R entered as a covariate was a decrease in variance explained of only 3%. Considering the main analyses of interest, SOI-R scores did not significantly impact the main effect of Sex [*F*(1, 243) = 31.13, *p* < 0.001, η^2^ = 0.11], the 2-way interaction of Sex and STI Contraction Likelihood on Sexual Engagement Likelihood [*F*(4, 240) = 7.85, *p* < 0.001, η^2^ = 0.12] or the 3-way interaction of Sex, STI Contraction Likelihood, and STI Type on Sexual Engagement Likelihood [*F*(12, 232) = 3.32, *p* < 0.001, η^2^ = 0.15]. See Supplementary Table 2 for all results.

### Secondary Analysis

While most participants indicated some level of sexual engagement likelihood across the different scenarios, 20 participants (17 females and 3 males) provided 0% likelihood of sexual engagement across all conditions (i.e., they would not have sex with the hypothetical partner under any circumstance). Since the higher proportion of females (85%) indicating 0% likelihood across the board might affect the results of the overall analysis, we deleted participants (both male and female) who indicated 0% likelihood of sexual engagement in all conditions and re-ran the analyses. As long as participants indicated having some likelihood of sexual engagement higher than 0% in any condition, they were retained in the analysis. Results indicated that this did not impact the findings in any considerable way. The 4-way interaction of Sex, STI Contraction Likelihood, STI Type, and Attractiveness Level on Sexual Engagement Likelihood remained significant [*F*(12, 213) = 2.06, *p* = 0.021, η^2^ = 0.10] as did the 3-way interaction of Sex, STI Contraction Likelihood, and STI Type on Sexual Engagement Likelihood [*F*(12, 213) = 4.41, *p* < 0.001, η^2^ = 0.20], and the 2-way interaction of Sex and STI Contraction Likelihood on Sexual Engagement Likelihood [*F*(4, 221) = 9.76, *p* < 0.001, η^2^ = 0.15] and the main effect of Sex on Sexual Engagement Likelihood [*F*(1, 224) = 41.82, *p* < 0.001, η^2^ = 0.16]. See Supplementary Table 3 for all results.

### Additional Analyses

Several variables were collected to investigate their impact on the main findings of the study. Previous Sex Education Course (yes or no), Religious Affiliation (yes or no), Currently Sexually Active (yes or no), Number of Sexual Partners, and Previous STI (yes or no) were each entered into the model as covariates and run separately. None of these variables significantly impacted the results of the targeted analyses reported above. Of the other demographic variables collected, Age and Relationship Status were found to be significantly different between males and females (see Table 1), but these variables were also entered as covariates and neither significantly impacted the results of the targeted analyses.

## Discussion

The present study investigated how male and female attitudes toward casual sexual encounters would change considering previously unexplored sexual risk-taking scenarios; STI contraction likelihoods and different STI types. We also assessed how partner attractiveness levels would shift sexual engagement likelihoods, and the role of participant sociosexuality. The results support many of our predictions. Overall, males reported significantly higher sexual engagement likelihoods compared to females, even though we took several steps to mitigate this effect (see below). This supports previous work showing male’s tendency to prefer sexual variety under short-term mating conditions (Clark and Hatfield, 1989; Clark, 1990; Kenrick et al., 1990; Simpson and Gangestad, 1992; Buss and Schmitt, 1993; Schmitt, 2003; Guéguen, 2011; Frederick et al., 2019). Males also reported significantly higher sexual engagement likelihoods across all five STI contraction likelihood categories compared to females, collapsed across STI type and attractiveness level (see Figure 1), reflecting male’s tendency to incur more risk for short-term mating opportunities in general.

The significant three-way interaction of Sex, STI contraction likelihood, and STI Type also indicated that males reported significantly higher sexual engagement likelihoods compared to females at lower STI contraction likelihoods (e.g., 5, 25%) for all four STI types (Cold, Herpes, Chlamydia, HIV), but both sexes were more similar in their responses as the likelihood of contracting an STI increased (e.g., 50, 100%), particularly for the more severe-rated STI types, Herpes and HIV (see Figure 2). We predicted that males would only consider a certain amount of risk under these hypothetical scenarios compared to females, thus the data supports our predictions. These effects remained unchanged even when removing participants (mostly females) who indicated 0% sexual engagement likelihoods for all scenarios.

Considering the attractiveness level of the hypothetical sex partner, the significant four-way interaction showed that sexual engagement likelihoods were impacted by each variable; Sex, STI Type, STI Contraction Likelihood, and Attractiveness Level. At lower STI contraction likelihoods (e.g., 5, 25%) less severe STI types (e.g., Cold, Chlamydia), and higher attractiveness levels (10 on a 1–10 scale), males continued to show higher sexual engagement likelihoods compared to females, but as STI contraction likelihoods increased, STI type severity increased, and attractiveness level decreased, sex differences in sexual engagement likelihood decreased. Planned comparison tests investigating sex differences for the two different attractiveness conditions showed that for the highest partner attractiveness condition (a 10), males had higher sex engagement likelihoods than females for all contraction likelihoods for the Common Cold, up to 50% contraction likelihood for Chlamydia, up to 25% contraction likelihood for Herpes, and 5% contraction likelihood for HIV. Fewer differences were found for the lower attractiveness condition (a 7) (see Supplementary Table 1). While the attractiveness level of the hypothetical partner significantly impacted sexual engagement likelihoods, it was not central to our investigation and had the smallest impact in the study. This may have been due to the differences we used for the attractiveness level condition; being a 7 or 10 on a 1–10 scale. For example, other work used larger differences to investigate the effect (e.g., the most attractive individual compared to someone with an average attractiveness; Guéguen, 2011, or using slight, moderately, and exceptionally attractive conditions, Schützwohl et al., 2009). Previous work shows that males are less concerned with mate quality characteristics under short-term mating conditions (Buss and Schmitt, 1993; Surbey and Conohan, 2000). Males have been shown to shift their willingness to pursue short-term mating encounters depending upon attractiveness, specifically lower attractiveness levels will decrease male likelihoods (Surbey and Conohan, 2000; Schützwohl et al., 2009). Our work complements these findings, as higher attractiveness levels increased the sex differences found in the study. In the context of risk-taking, the data also supports other work (Baker and Maner, 2008; Ronay and von Hippel, 2010) which showed that the presence of an attractive female increases risk-taking among males. Based on our findings, males seem to be more willing to incur costs associated with STIs to have sexual access with more attractive females.

Within each variable condition, we assessed the likelihood of sexual engagement even when contracting an STI would not happen (0% likelihood). Males were more likely to indicate they would engage in a casual sexual encounter in this condition compared to females overall. We took several steps to mitigate potential confounds that might hinder a female’s propensity to engage in a short-term mating encounter by incorporating information about pregnancy, sexual gratification, and social context into our hypothetical scenario (Conley et al., 2011; Pedersen et al., 2011; Galperin et al., 2013; Kennair et al., 2016). This additional information did little to change sex differences in casual sex engagement likelihoods. As such, Kennair et al. (2016) found that potential negative consequences (e.g., pregnancy) and physical gratification of casual sex did not account for sex differences in their study, which considered sexual regret. Kennair et al. (2016) also found that females worried more about STIs than males. Coupled with the fact that males are estimated to be at double the risk of contracting STIs (Courtenay, 2000), it seems clear that males are willing to put themselves at risk for the opportunity to have casual sex. This comports with previous work (Clark, 1990) who found similar sex differences in casual sex during the AIDS epidemic.

### SOI-R Results

While entering SOI-R values into the model as a covariate did decrease sex differences to some extent, it did not do so in a substantial way. The 2-way interaction of Sex and STI Contraction Likelihood continued to explain 12% of the variance in Sexual Engagement Likelihoods, a decrease of 5% from the original analysis, and the 3-way interaction of Sex, STI Contraction Likelihood, and STI Type continued to explain 15% of the variance in Sexual Engagement Likelihoods, a decrease of 6% from the original analysis. Although the 4-way interaction of Sex, STI Contraction Likelihood, STI Type, and Attractiveness Level on Sexual Engagement Likelihoods did result in a non-significant outcome, it only decreased the percentage of variance explained in the model by 3% from the original analysis. These results indicate that although the sociosexuality of participants plays a role in attitudes toward casual sex, it did not negate the sex differences in attitudes toward casual sex that were originally found. Previous work (Hallam et al., 2018) showed that by controlling for SOI-R scores, sex differences toward sexual motivation can be eliminated. While sociosexuality clearly plays a role in attitudes toward casual sex, our findings suggest that it does not negate the sex differences. Other work (Kennair et al., 2016) questions whether including SOI-R values as a covariate is justified when investigating sex differences, since different levels of sociosexuality are inherently a part of what constitutes being male or female in general. We, along with others (e.g., Schmitt, 2005; Penke and Asendorpf, 2008; Lippa, 2009; Kennair et al., 2016; Nascimento et al., 2018), found that males have higher levels of sociosexuality compared to females. Therefore, concluding that there are no sex differences in a study that controls for sociosexuality might be short-sighted (Schmitt et al., 2012). However, it is interesting to see how variables impact sex attitudes, thus we can see the impetus for researchers to include the variable in their work.

### Additional Analyses

Additional analyses were conducted which removed participants that were not willing to engage in any casual sex encounter under any circumstance (85% of these 20 participants were female). This discrepancy between males and females was expected since past work shows females are more discriminate considering casual sex opportunities overall (Buss and Schmitt, 1993; Buss, 2015). The removal of these participants did little to change the findings of the targeted analyses in the study (see Supplementary Table 3).

Since sexual education among adolescents can impact STI knowledge and subsequent attitudes toward sex (Widman et al., 2018) we assessed whether there were any sex differences in exposure to sex education. Findings showed that males and females were no different in sexual education access and it did not impact any significant effects when entered into the model as a covariate. We had participants rank the STIs used in the study on levels of severity and found a high rate of consensus among rankings overall, also between males and females (see Supplementary Table 4). We also investigated whether sex differences in religious affiliation and being sexually active would impact the results; Neither variable was found to impact the findings in a meaningful way. Of the other demographic variables collected in the study, Age and Relationship Status were found to be significantly different between males and females. However, none of these variables when entered as covariates significantly impacted the overall results of the targeted analyses. In conjunction with our attempt to limit biases that would hinder females from engaging in casual sex scenarios, these results provide evidence for the sex differences found in this study to reflect male’s willingness to incur more risk considering short-term mating encounters.

### Limitations

Our study was based on hypothetical scenarios which may not reflect actual mating decisions. While this is a potential weakness, hypothetical scenarios in research are not uncommon (especially when studying intimate human behaviors). In a meta-analysis of risk-taking behaviors, a quarter of surveyed experiments and half of dissertations used hypothetical choice scenarios (Byrnes et al., 1999). Regarding sexual risk-taking, having protected sex is strongly related to subjects’ intentions to do so (Reinecke et al., 1996) and attitude-behavioral congruence has been established investigating extra marital affairs (Glass and Wright, 1992). Additionally, several articles cited in this paper used hypothetical choices, rather than self-reported behavior (Kenrick et al., 1990; Rolison and Scherman, 2003; Li and Kenrick, 2006; Lippa, 2009). Outside of collecting previous mating encounters, hypothetical scenarios provide a great window into the evolved mind. Symons (1979) notes that the function of the mind is to cause behavioral action, even for events that have a low probability of occurring. Investigating sexual motivation under specific situations provides researchers with a better understanding of future behavioral action should those events unfold.

Another potential limitation of our results is the makeup of our sample: Participants were younger college students, with 64% identifying as White/Caucasian. While our findings might not generalize to other populations perfectly, previous work investigating sex differences in sex attitudes/behavior across the globe show similar trends (Buss, 1989; Schmitt, 2003, 2005; Lippa, 2009). Therefore, our work might not be limited to only college students, although future studies will help clarify this potential issue.

## Conclusion

Previous work has shown that males take more risks in a variety of contexts. This is thought to be a consequence of males’ evolved tendency to be more opportunistic in their mating strategies compared to females (Buss and Schmitt, 1993; Schmitt, 2003). The present findings complement this perspective and show that males are more willing to incur risks associated with STI contraction when considering short-term sexual encounters. We took several steps in an attempt to limit any biases that would hinder females to pursue short-term mating encounters in the first place, and we also statistically assessed whether several other variables impacted the findings. Our results also remain robust after controlling for participants’ level of sociosexuality. Although males and females both utilize long- and short-term mating strategies (Gangestad and Simpson, 2000; Buss and Schmitt, 2019), males, if given the opportunity exhibit mating decisions that reflect higher sexual risk compared to female’s more discriminant mating propensities.

## Data Availability Statement

The raw data supporting the conclusions of this article will be made available by the authors, without undue reservation.

## Ethics Statement

The studies involving human participants were reviewed and approved by the Florida Gulf Coast University Institutional Review Board. The patients/participants provided their written informed consent to participate in this study.

## Author Contributions

RP, LC, MK, FV, and SM made substantial contributions to the study conception, design, and IRB protocol. LC, HM, DA, SS, MK, FV, SM, and EC collected the data. RP analyzed the data. All authors contributed to the drafting and revising of the article and approved the final version of the manuscript.

## Conflict of Interest

The authors declare that the research was conducted in the absence of any commercial or financial relationships that could be construed as a potential conflict of interest.

## Publisher’s Note

All claims expressed in this article are solely those of the authors and do not necessarily represent those of their affiliated organizations, or those of the publisher, the editors and the reviewers. Any product that may be evaluated in this article, or claim that may be made by its manufacturer, is not guaranteed or endorsed by the publisher.

## Footnotes

1
https://www.sona-systems.com/
