# Supplementary material for: Sex Differences in Attitudes Toward Casual Sex: Using STI Contraction Likelihoods to Assess Evolved Mating Strategies
Source: Front Psychol. 2021 Sep 3;12:706149. doi: 10.3389/fpsyg.2021.706149 (PMC8446665; doi:10.3389/fpsyg.2021.706149)
Supplement: Supplementary file 1 [file Data_Sheet_1.docx]

**Disease descriptions**

**Common Cold**

A viral infection of your nose and throat (upper respiratory tract). Symptoms for a common cold include a runny nose, sore throat, and cough. Treatment typically consists of pain relievers, decongestant nasal sprays, and cough syrups. If left untreated, may lead to inflammation and infection of the sinuses (sinusitis).

**Herpes**

A common virus contracted by engaging in vaginal, anal, or oral sex with someone who has the disease. Mild symptoms include sores, which appear as one or more blisters around the genitals, rectum, or mouth. In rare cases, herpes can be transmitted from physical contact. There is no cure for this infection, but treatment consists of medication to reduce outbreaks and prevent transmission of virus. If left untreated, painful genital sores can occur and sores can be transmitted to other body parts through fluid contact.

**Chlamydia**

An infection caused by the bacteria Chlamydia trachomatis, is contracted by engaging in vaginal, anal, or oral sex with someone who has the infection. Most people who contract chlamydia do not suffer any symptoms but those who do often suffer from pain during urination and discharge from genitals. Chlamydia can be cured through antibiotics. Antibiotics will stop the infection but will not undo any permanent damage. If left untreated, chlamydia can cause pelvic inflammatory disease and infertility in women. In rare cases, chlamydia can cause infertility in men.

**HIV**

A virus that spreads through certain body fluids that attack the body’s immune system. Symptoms for HIV include flu-like illness within 2 to 4 weeks after infection. Although there is no cure for this infection, treatment typically consists of Antiretroviral therapy, which are medications that may slow the progression of the virus in the body. If left untreated, HIV may lead to AIDS. AIDS is the most severe phase of the HIV infection. People with AIDS have a severely damaged immune system, which often leads to a shortened life span.
