# Supplementary material for: Sex Differences in Attitudes Toward Casual Sex: Using STI Contraction Likelihoods to Assess Evolved Mating Strategies
Source: Front Psychol. 2021 Sep 3;12:706149. doi: 10.3389/fpsyg.2021.706149 (PMC8446665; doi:10.3389/fpsyg.2021.706149)
Supplement: Supplementary file 2 [file Table_1.docx]

Table S1. A-priori planned comparisons using independent sample *t*-tests investigated the extent to which sex differences exist in Sexual Engagement Likelihoods as STI Contraction Likelihoods increased. Tests were run for each of the four STI Types and for both Attractiveness Level conditions (7 or 10 and a 1-10 scale).

| Variable | Males | Females | *t*-value | *p* |
| --- | --- | --- | --- | --- |
| Common Cold |  |  |  |  |
| Attractiveness 7 |  |  |  |  |
| 0% Contraction | 75.10 (29.50) | 48.52 (33.80) | 6.58 | <.001* |
| 5% Contraction | 63.02 (32.53) | 34.74 (30.34) | 7.04 | <.001* |
| 25% Contraction | 49.75 (31.89) | 25.87 (26.76) | 6.38 | <.001* |
| 50% Contraction | 33.12 (30.47) | 14.84 (20.51) | 5.40 | <.001* |
| 100% Contraction | 12.93 (23.40) | 5.14 (12.94) | 3.13 | .002* |
| Common Cold |  |  |  |  |
| Attractiveness 10 |  |  |  |  |
| 0% Contraction | 85.39 (27.32) | 59.99 (36.75) | 6.21 | <.001* |
| 5% Contraction | 74.98 (33.12) | 45.46 (34.43) | 6.81 | <.001* |
| 25% Contraction | 63.29 (35.61) | 33.76 (30.51) | 7.01 | <.001* |
| 50% Contraction | 50.97 (36.21) | 21.29 (25.71) | 7.26 | <.001* |
| 100% Contraction | 34.66 (37.60) | 12.09 (22.33) | 5.57 | <.001* |
| Herpes |  |  |  |  |
| Attractiveness 7 |  |  |  |  |
| 0% Contraction | 72.32 (33.71) | 42.79 (35.56) | 6.67 | <.001* |
| 5% Contraction | 22.50 (27.05) | 11.39 (22.84) | 3.44 | .001* |
| 25% Contraction | 9.37 (16.55) | 6.01 (14.96) | 1.67 | .096 |
| 50% Contraction | 3.01 (8.57) | 2.83 (10.52) | .15 | .885 |
| 100% Contraction | .45 (3.54) | 1.12 (9.59) | -.67 | .487 |
| Herpes |  |  |  |  |
| Attractiveness 10 |  |  |  |  |
| 0% Contraction | 83.02 (30.09) | 52.68 (38.70) | 6.92 | <.001* |
| 5% Contraction | 34.68 (36.04) | 15.58 (27.96) | 4.57 | <.001* |
| 25% Contraction | 15.41 (24.59) | 7.81 (18.80) | 2.68 | .008* |
| 50% Contraction | 5.79 (13.85) | 3.30 (11.65) | 1.51 | .132 |
| 100% Contraction | 1.01 (5.651) | 1.11 (7.40) | -.12 | .905 |
| Chlamydia |  |  |  |  |
| Attractiveness 7 |  |  |  |  |
| 0% Contraction | 73.56 (31.92) | 41.27 (36.51) | 7.40 | <.001* |
| 5% Contraction | 27.73 (32.74) | 10.87 (22.83) | 4.59 | <.001* |
| 25% Contraction | 12.88 (22.40) | 5.23 (13.781) | 3.14 | .002* |
| 50% Contraction | 4.23 (10.64) | 1.95 (7.64) | 1.89 | .060 |
| 100% Contraction | .63 (3.35) | .00 (.00) | 1.98 | .050 |
| Chlamydia |  |  |  |  |
| Attractiveness 10 |  |  |  |  |
| 0% Contraction | 82.05 (31.69) | 51.05 (39.44) | 6.84 | <.001* |
| 5% Contraction | 34.96 (36.90) | 15.53 (28.39) | 4.55 | <.001* |
| 25% Contraction | 16.32 (26.09) | 7.17 (17.31) | 3.16 | .002* |
| 50% Contraction | 5.93 (15.83) | 2.32 (8.305) | 2.17 | .032* |
| 100% Contraction | 1.31 (9.76) | .74 (6.06) | .56 | .579 |
| HIV |  |  |  |  |
| Attractiveness 7 |  |  |  |  |
| 0% Contraction | 72.24 (34.29) | 40.97 (36.22) | 6.94 | <.001* |
| 5% Contraction | 12.50 (24.58) | 6.99 (18.91) | 1.94 | .054 |
| 25% Contraction | 4.03 (12.79) | 3.43 (11.71) | .38 | .703 |
| 50% Contraction | 1.13 (5.16) | 1.47 (6.54) | -.45 | .656 |
| 100% Contraction | .04 (.38) | .74 (6.06) | -1.35 | .180 |
| HIV |  |  |  |  |
| Attractiveness 10 |  |  |  |  |
| 0% Contraction | 80.91 (33.04) | 49.93 (40.43) | 6.61 | <.001* |
| 5% Contraction | 16.68 (28.71) | 7.68 (20.42) | 2.78 | .006* |
| 25% Contraction | 4.25 (13.39) | 3.67 (12.39) | .35 | .726 |
| 50% Contraction | 1.23 (6.03) | 1.28 (6.05) | -.07 | .942 |
| 100% Contraction | .10 (.81) | .37 (4.30) | -.65 | .513 |

Note. Mean Engagement Likelihoods are provided with standard deviations in parentheses.

**p* < .05 is considered statistically significant.
