# Supplementary material for: Sex Differences in Attitudes Toward Casual Sex: Using STI Contraction Likelihoods to Assess Evolved Mating Strategies
Source: Front Psychol. 2021 Sep 3;12:706149. doi: 10.3389/fpsyg.2021.706149 (PMC8446665; doi:10.3389/fpsyg.2021.706149)
Supplement: Supplementary file 3 [file Table_2.docx]

Table S2. All GLM mixed-model ANCOVA main effects and 2-way, 3-way, and 4-way interactions of Sex, STI Contraction Likelihood, STI Type, and Attractiveness Level on the dependent variable, Sexual Engagement Likelihood, with SOI-R scores entered as a covariate. Bolded results represent targeted analyses.

| Independent Variables | *df_1_, df_2_* | *f* | *p* | *η^2^* |
| --- | --- | --- | --- | --- |
| **Sex** | **1, 243** | **31.13** | **< .001** | **.11** |
| STI Contraction Likelihood | 4, 240 | 6.32 | < .001 | .10 |
| STI Type | 3, 241 | .41 | .743 | .005 |
| Attractiveness Level | 1, 243 | .082 | .744 | <.001 |
| **Sex * STI Contraction Likelihood** | **4, 240** | **7.85** | **<.001** | **.12** |
| Sex * STI Type | 3, 241 | 8.69 | <.001 | .10 |
| Sex * Attractiveness Level | 1, 243 | 4.35 | .038 | .02 |
| STI Type * Attractiveness Level | 3, 241 | 1.34 | .264 | .02 |
| STI Contraction Likelihood * STI Type | 12, 232 | 1.46 | .14 | .07 |
| STI Contraction Likelihood * Attractiveness Level | 4, 240 | 1.83 | .124 | .03 |
| **Sex * STI Contraction Likelihood * STI Type** | **12, 232** | **3.32** | **<.001** | **.15** |
| Sex * STI Type * Attractiveness Level | 3, 241 | 2.22 | .086 | .03 |
| Sex * STI Contraction Likelihood * Attractiveness Level | 4, 240 | 2.54 | .041 | .04 |
| STI Contraction Likelihood * STI Type * Attractiveness Level | 12, 232 | .91 | .542 | .05 |
| **Sex * STI Contraction Likelihood * STI Type * Attractiveness Level** | **12, 232** | **1.58** | **.097** | **.08** |

Note: STI = Sexually Transmitted Infection, *df_1_* = numerator degrees of freedom, *df_2_* = denominator degrees of freedom, *f* = multivariate Pillai’s trace value, *p* = significance value (*p* < .05 considered significant), *η^2^* = partial eta-squared.
