# Supplementary material for: Sex Differences in Attitudes Toward Casual Sex: Using STI Contraction Likelihoods to Assess Evolved Mating Strategies
Source: Front Psychol. 2021 Sep 3;12:706149. doi: 10.3389/fpsyg.2021.706149 (PMC8446665; doi:10.3389/fpsyg.2021.706149)
Supplement: Supplementary file 4 [file Table_3.docx]

Table S3. All mixed-model ANOVA main effects and 2-way, 3-way, and 4-way interactions of Sex, STI Contraction Likelihood, STI Type, and Attractiveness Level on the dependent variable, Sexual Engagement Likelihood, with participants who responded with 0% likelihood in all conditions removed (17 females, 3 males). Bolded results represent targeted analyses.

| Independent Variables | *df_1_, df_2_* | *f* | *p* | *η^2^* |
| --- | --- | --- | --- | --- |
| **Sex** | **1, 224** | **41.82** | **<.001** | **.16** |
| STI Contraction Likelihood | 4, 221 | 267.23 | < .001 | .83 |
| STI Type | 3, 222 | 153.21 | <.001 | .67 |
| Attractiveness Level | 1, 224 | 202.54 | <.001 | .48 |
| **Sex * STI Contraction Likelihood** | **4, 221** | **9.76** | **<.001** | **.15** |
| Sex * STI Type | 3, 222 | 12.53 | <.001 | .15 |
| Sex * Attractiveness Level | 1, 224 | 8.37 | .004 | .04 |
| STI Type * Attractiveness Level | 3, 222 | 49.67 | <.001 | .40 |
| STI Contraction Likelihood * STI Type | 12, 213 | 32.22 | <.001 | .65 |
| STI Contraction Likelihood * Attractiveness Level | 4, 221 | 18.56 | <.001 | .25 |
| **Sex * STI Contraction Likelihood * STI Type** | **12, 213** | **4.41** | **<.001** | **.20** |
| Sex * STI Type * Attractiveness Level | 3, 222 | 4.46 | .005 | .06 |
| Sex * STI Contraction Likelihood * Attractiveness Level | 4, 221 | 3.32 | .011 | .06 |
| STI Contraction Likelihood * STI Type * Attractiveness Level | 12, 213 | 5.641 | <.001 | .24 |
| **Sex * STI Contraction Likelihood * STI Type * Attractiveness Level** | **12, 213** | **2.06** | **.021** | **.10** |

Note: STI = Sexually Transmitted Infection, *df_1_* = numerator degrees of freedom, *df_2_* = denominator degrees of freedom, *f* = multivariate Pillai’s trace value, *p* = significance value (*p* < .05 considered significant), *η^2^* = partial eta-squared.
