# Supplementary material for: Sex Differences in Attitudes Toward Casual Sex: Using STI Contraction Likelihoods to Assess Evolved Mating Strategies
Source: Front Psychol. 2021 Sep 3;12:706149. doi: 10.3389/fpsyg.2021.706149 (PMC8446665; doi:10.3389/fpsyg.2021.706149)
Supplement: Supplementary file 5 [file Table_4.docx]

Table S4. STI Severity rankings by participants in the study.

| STI | Least Severe | Second Least Severe | Second Most Severe | Most Severe |
| --- | --- | --- | --- | --- |
| Common Cold | 92% | 7% | 0% | 1% |
| Chlamydia | 5% | 47% | 41% | 7% |
| Herpes | 2% | 44% | 53% | 2% |
| HIV | 2% | 2% | 6% | 90% |

Note: STI = Sexually Transmitted Infection. No significant differences were detected between males and females for each of the STI variables, all *X*^2^ values < 6.5, *p’s* > .09
